# Utility of non‐contact thermometers in neonatal intensive care: Effects of incubator conditions and measurement sites

**DOI:** 10.1111/ped.70231

**Published:** 2025-10-14

**Authors:** Takashi Okuno, Tatsuto Shimizu, Aiko Igarashi, Yusei Ohshima

**Affiliations:** ^1^ Department of Pediatrics, Faculty of Medical Sciences University of Fukui Fukui Japan

**Keywords:** body temperature regulation, incubator, infrared thermometer, newborn, temperature measurement

## Abstract

**Background:**

Non‐contact infrared thermometers (NCITs) are non‐invasive alternatives to conventional contact thermometers (CTs). However, the accuracy of NCITs may be affected by measurement environments and body surface sites. This study evaluated the reliability of NCIT measurements under various temperature conditions in neonatal incubators and explored optimal measurement strategies.

**Methods:**

Preterm neonates with a corrected gestational age of 25–32 weeks were enrolled in this observational study. Body temperature was measured using NCITs at the axilla, forehead, and both contact and non‐contact surfaces of the trunk, and by CT in the axilla. Measurement environments were classified into two groups: high temperature (HT [≥33°C, ≥60% humidity]) and low temperature (LT [28.5–32.9°C, ≥40% humidity]).

**Results:**

Body temperatures measured by NCIT correlated with axillary temperature measured by CT (axillary CT) but varied by site. The non‐contact trunk surface measured by NCIT had the best correlation with temperature measured by axillary CT and had the smallest limits of agreement in the HT group. However, Bland–Altman analysis showed wide limits of agreement for single‐site NCITs in the LT group, although the correlation at the contact trunk surface improved with corrected gestational age. Averaging NCIT readings from the axilla and both trunk surfaces yielded stronger correlations with axillary CT (*p* < 0.05).

**Conclusions:**

NCITs appear to be a practical option for neonatal temperature monitoring, particularly when used at trunk surface sites. Taking average measurements from multiple sites may enhance accuracy and aid in creating better measurement protocols in NICUs.

## INTRODUCTION

Effective temperature management in neonatal intensive care units (NICUs) is crucial for the survival and well‐being of neonates. Preterm and very low birth weight (VLBW) infants are particularly vulnerable to hypo‐ and hyperthermia due to immature thermoregulatory systems, making precise temperature monitoring an essential component of neonatal care.^1^, ^2^, ^3^ In addition to the physiological importance, frequent interventions required for temperature monitoring in NICUs can cause significant stress and discomfort for neonates. Such distress may contribute to long‐term developmental and behavioral consequences.^4^, ^5^, ^6^

Conventional contact thermometers (CTs), while accurate, can be distressing to use and may increase the risk of infection, highlighting the need for non‐invasive, rapid, and infection‐free alternatives.^7^, ^8^ Non‐contact infrared thermometers (NCITs) have emerged as a promising solution, offering quick and non‐invasive temperature assessment.^9^, ^10^, ^11^ Studies have demonstrated the reliability of NCITs in healthy and preterm neonates and show high agreement with CT readings within clinically acceptable limits. However, studies evaluating NCIT reliability in NICU settings have yielded inconsistent results. Sollai et al. reported that NCITs provide temperature measurements comparable to axillary thermometry.^9^ Placidi et al. examined the influence of measurement sites on temperature readings and highlighted the variability across different body regions.^12^ Robertson‐Smith et al. reported that mid‐forehead temperatures measured with NCITs exhibited high variability and a low correlation with axillary temperatures, particularly in neonates receiving respiratory support, suggesting that NCITs may not be suitable for all NICU conditions.^13^

Despite the potential, the accuracy of NCITs under diverse clinical conditions, especially within incubators, has not been thoroughly studied. Environmental factors, such as incubator temperature and humidity, are known to influence measurement accuracy, yet the impact in clinical settings has not been established.^7^, ^8^, ^10^ Furthermore, existing research has not adequately addressed the variability in NCIT readings across different anatomic sites, such as the forehead, chest, and back. Additionally, strategies to minimize stress associated with temperature monitoring in neonates have not been studied.^4^, ^6^

These gaps in knowledge underscore the need for robust investigations to establish evidence‐based guidelines for integrating NCITs into routine NICU practices. The current study aimed to assess the utility of NCITs under varying incubator conditions by comparing axillary temperature measurements obtained with CTs to multi‐site NCIT readings. By addressing these gaps, we sought to improve neonatal temperature monitoring protocols, reduce stress and infection risks associated with invasive procedures, and enhance overall NICU care.

## METHODS

### Study design

This was a single‐center, observational study conducted in the NICU at the University of Fukui Hospital. The primary objective was to assess the agreement and variability of measurements taken with an NCIT under different incubator temperature conditions. Ethical approval was obtained from the Institutional Review Board (IRB) of the University of Fukui Hospital (approval number: 20240177). The study adhered to internationally recognized ethical standards, including the Declaration of Helsinki. Because this was a retrospective observational study, the requirement for informed consent was waived by the IRB of Fukui University Hospital.

### Study participants

**FIGURE 1:**
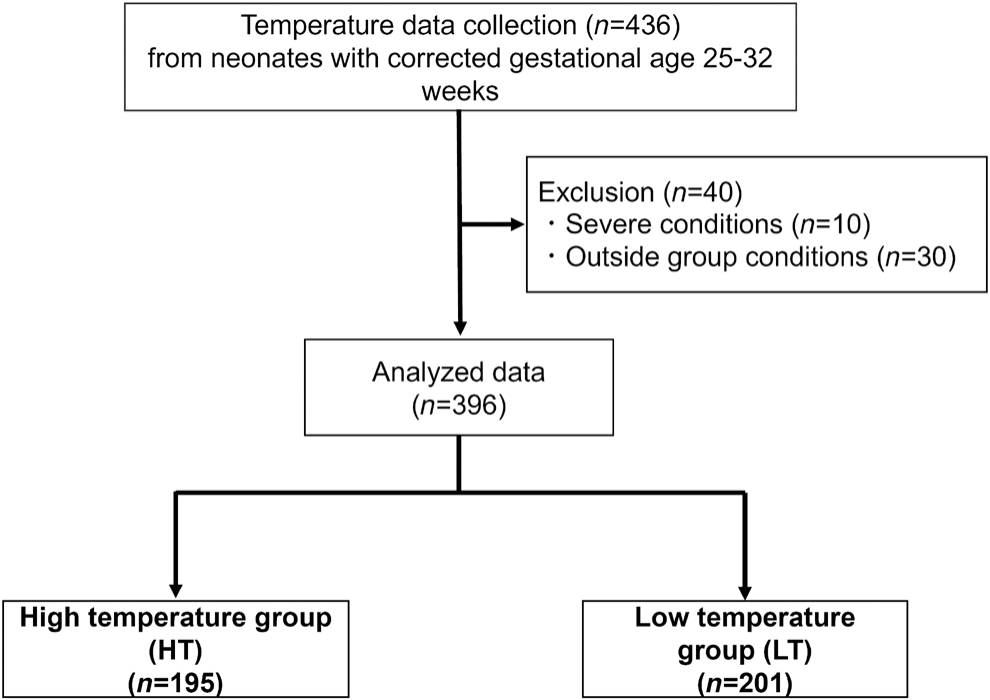
Study participant flow. Neonates admitted to the NICU with a postnatal age of 0–25 days, a corrected gestational age of 25–33 weeks, and a birth weight of <1500 g were included in the study. Among the 436 temperature measurements performed, temperatures obtained under critical conditions (*n* = 10) and temperatures obtained in incubator environments that did not meet the criteria for either the HT or LT group (*n* = 30) were excluded. As a result, the remaining measurements were classified into HT (*n* = 195) and LT (*n* = 201).

The current study included neonates admitted to the NICU at the University of Fukui Hospital between 1 December 2022 and 30 November 2024. The inclusion criteria were neonates with a corrected gestational age of 25–32 weeks who required thermal and humidity management in an incubator. Neonates with unstable medical conditions (e.g., cardiovascular instability or severe congenital anomalies [*n* = 10]) or who did not meet group inclusion criteria (*n* = 30) were excluded, as shown in Figure 1. Ultimately, 20 neonates were enrolled in the current study. None of the enrolled neonates had skin conditions that interfered with temperature measurements. The median gestational age was 27.5 weeks (range: 25–30 weeks) and the median birth weight was 1014.5 g (range: 420–1682 g). The study cohort included 11 male and 9 female neonates. Four neonates were delivered vaginally and 16 were delivered via cesarean section. A total of 436 temperature measurements were initially collected. After excluding 40 measurements due to predefined criteria, 396 measurements were included in the final analysis (Figure 1).

### Temperature and humidity conditions of the incubator

Participants were categorized into two groups based on incubator temperature and humidity conditions. Under high‐temperature conditions (HT), incubators were set at temperatures ≥33°C and humidity levels ≥60% because previous studies have suggested that extremely preterm neonates require higher humidity and temperature to support thermoregulation.^14^, ^15^ The initial incubator temperature for very low birth weight infants in our NICU is set at 33°C and was used as the cut‐off between the HT and low‐temperature conditions (LT) groups. In contrast, the LT group included incubators with temperatures between 28.5°C and 32.9°C and humidity levels ≥40%, reflecting conditions commonly observed in NICUs for more mature neonates. Temperature and humidity data were recorded using the integrated monitoring system of a closed incubator (Dual Incu I; Atom Medical Corporation, Tokyo, Japan [approval number: 22200BZX00149000]).

### Measurement procedure

Body temperature measurements were obtained for each neonate according to a standardized protocol. Measurements were performed 4–8 times per day. All NCIT measurements were performed at a standardized distance of 2–3 cm from the skin to ensure accuracy, as recommended by the manufacturer. Measurements were taken at five anatomic sites using a CT or an NCIT.

**FIGURE 2:**
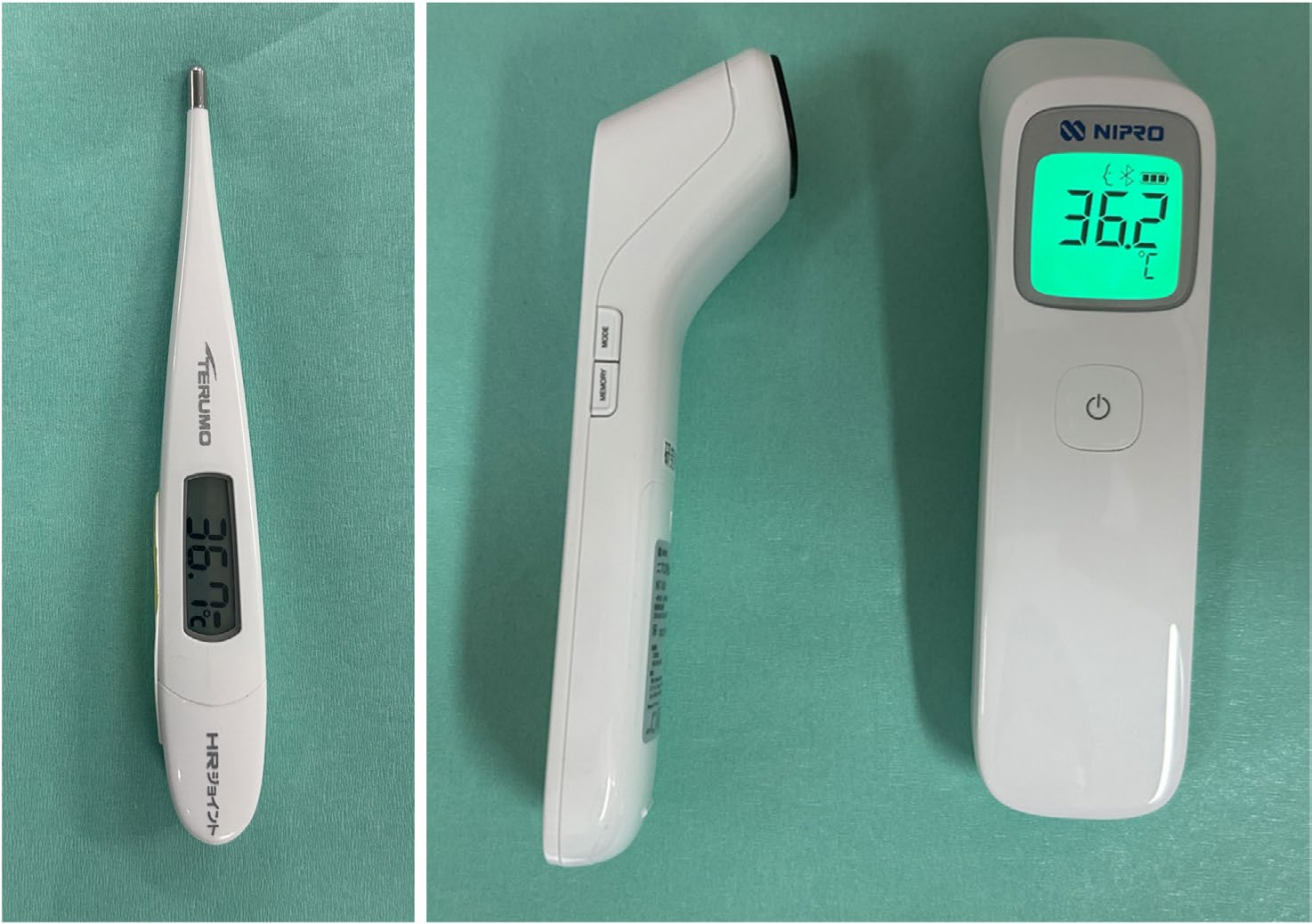
Thermometers used in the study. This study used contact (left) and non‐contact thermometers (right).

The axillary temperature using a CT (axillary CT) was measured using an electronic axillary thermometer (ET‐C207P; Terumo Corporation, Tokyo, Japan [approval number: 302AABZX00003000]), which provides predictive readings within approximately 30 sec. Axillary and forehead temperatures were measured using an NCIT (NT‐100B; Nipro Corporation, Osaka, Japan [approval number: 303AABZX00042000]) at the axilla and the center of the forehead, respectively (Figure 2).

Trunk temperatures were recorded at two distinct sites to assess the impact of contact with the incubator surface. The non‐contact trunk surface temperature was measured on the side of the trunk that was not in direct contact with the incubator or bedding; specifically, the measurement sites were the chest in the supine position or the back in the prone position. In contrast, the contact trunk surface temperature was measured on the surface of the trunk that was in direct contact with the incubator or bedding; specifically, the measurement sites were the back in the supine position or the chest in the prone position, which ensured that the skin was in direct contact with the measurement surface before recording the temperature. Both of these trunk surface measurements were taken using the same NCIT; the difference between “contact” and “non‐contact” refers only to whether the skin touches the bedding, not to the measurement technique. The contact trunk surface temperature was measured during routine care for neonates requiring a fixed position (supine or prone) for medical reasons, such as pressure relief or clinical observation, to avoid additional stress.

The posture of each neonate (supine or prone) was recorded during every measurement. In addition, measurements were performed by 25 NICU staff members with 1–20 years of clinical experience.

### Outcome measures

The primary outcome of this study was the correlation and agreement between body temperature measured by NCIT and axillary CT. Secondary outcomes included an assessment of measurement variability at each site and an evaluation of how incubator temperature and humidity conditions (HT vs. LT groups) influenced measurement accuracy and precision, the impact of infant maturity on temperature readings, and examiner‐dependent variability under consistent measurement conditions.

### Statistical analysis

Statistical analyses were performed using R software (version 4.3.1; R Foundation for Statistical Computing, Vienna, Austria). Data normality was assessed using the Shapiro–Wilk test and Q–Q plots. The correlation between NCIT and CT measurements was analyzed using Pearson's correlation coefficient.

A Bland–Altman analysis was performed to evaluate the agreement between the body temperature measured by NCIT and axillary CT. The analysis assesses agreement between two measurements using bias, standard deviation, and 95% limits of agreement (LoA); a wider LoA indicates greater variability. Differences among measurement sites were assessed using the Friedman test with adjustments made using the Bonferroni correction. Variability between temperature and humidity groups was compared using t‐tests. To compare correlations between single‐site measurements and multi‐site averages, Fisher's z‐transformation was applied.

Temperature bias, defined as the difference between the temperature measured by NCIT and axillary CT, was analyzed in relation to developmental maturity. One‐way ANOVA assessed the association with corrected gestational age (CGA) and two‐way ANOVA examined interactions between gestational and postnatal age. The HT and LT groups were stratified by CGA for further correlation and Bland–Altman analyses.

Examiner‐related variability was assessed using a linear mixed‐effects model with axillary CT as a fixed effect and examiner ID as a random intercept. Intraclass correlation coefficients (ICCs) quantified examiner‐related variance. A *p* value <0.05 was considered statistically significant.

## RESULTS

### Characteristics of patients and environment upon temperature measurement

A total of 396 temperature measurements were obtained, with 195 in the HT and 201 in the LT groups (Figure 1). Newborns in whom temperatures were measured in an HT environment (HT group) were significantly younger (postnatal age, 6 [range 0–22] days vs. 11 [range 1–25] days; corrected gestational age, 26 [range 25–29] weeks vs. 29 [range, 26–33] weeks) and had lower body weights (610 [range 554–948] g (HT) vs. 982 [range 763–1489] g) than newborns in whom temperatures were measured in an LT (LT group; Table 1). There was no difference in the time of day when the body temperature was measured between HT and LT groups. The percentage of newborns in the supine position before body temperature measurement was higher in the HT than in the LT group.

**TABLE 1:** Characteristics of the study subjects and conditions upon body temperature measurement.

	Overall (*n* = 395)	High temperature HT (*n* = 194)	Low temperature LT (*n* = 201)	*p* Value
Postnatal age (day)	9 [0, 25]	6 [0, 22]	11 [1, 25]	<0.05
Corrected GA (weeks)	27 [25, 33]	26 [25, 29]	29 [26, 33]	<0.05
Measured weight (g)	858 [554, 1489]	610 [554, 948]	982 [763, 1489]	<0.05
Incubator humidity (%)	72 [40, 84]	75 [60, 83]	61 [40, 84]	<0.05
Incubator temperature (°C)	32.8 [28.5 37.5]	35 [33, 37.5]	30.3 [28.5, 32.9]	<0.05
Measurement time *n* (%)				
AM	197 (49.9)	88 (45.4)	109 (54.2)	0.087
PM	198 (50.1)	106 (54.6)	92 (45.8)	
Respiratory support *n* (%)				
Intubation	194 (49.1)	188 (96.9)	6 (3.0)	<0.05
nCPAP	197 (49.9)	6 (3.1)	191 (95.0)	
O_2_ Therapy	4 (1.0)	0 (0.0)	4 (2.0)	
Pre‐position *n* (%)				
Supine	136 (34.4)	93 (47.9)	43 (21.4)	<0.05
Prone	259 (65.6)	101 (52.1)	158 (78.6)	

*Note*: Values in brackets represent the interquartile range (IQR).

Abbreviations: GA, gestational age.

### Effect of incubator environment on measured body temperature

The axillary CT was consistently lower than body temperatures measured by an NCIT (*p* < 0.05), irrespective of incubator setting. Although there was no significant difference in the axillary CT between the HT and LT groups, the temperature measured by an NCIT at each body surface site was significantly higher in the HT than the LT group (*p* < 0.001; Table 2). The contact trunk surface and forehead had the highest and lowest temperatures among sites measured by an NCIT in the LT group. The contact trunk surface also had the highest temperature among sites measured by an NCIT in the HT group, but the temperatures measured at other body surface sites were similar. The disparity between the highest and lowest body temperatures measured by an NCIT at nearly the same time was 0.59 ± 0.30°C in the HT group and 0.86 ± 0.42°C in the LT group, indicating a significantly larger disparity in the LT group compared to the HT group.

**TABLE 2:** Body temperature measured by CT and NCIT under different incubator settings.

	Overall (*n* = 395), mean (SD)	High temperature HT (*n* = 194), mean (SD)	Low temperature LT (*n* = 201), mean (SD)	*p* Value, HT vs. LT
Contact thermometer (CT)				
Axillary CT	37.12 (0.40)	37.09 (0.39)	37.15 (0.40)	0.155
Non‐contact infrared thermometer (NCIT)				
Axilla	37.89 (0.52)	38.08 (0.45)	37.70 (0.52)	<0.001
Forehead	37.81 (0.60)	38.13 (0.49)	37.50 (0.53)	<0.001
Non‐contact trunk	37.85 (0.53)	38.07 (0.48)	37.63 (0.49)	<0.001
Contact trunk	38.15 (0.55)	38.29 (0.52)	38.01 (0.54)	<0.001
NCIT max–min	0.73 (0.39)	0.59 (0.30)	0.86 (0.42)	<0.001

Abbreviations: CT, contact thermometer; NCIT, Non‐contact infrared thermometer.

### Correlation between body temperature measured by axillary CT and NCIT

Correlation and agreement were assessed using Pearson's correlation coefficient and Bland–Altman to identify NCITs that were useful as substitutes for the axillary CT. The temperature of the contact trunk surface had the highest correlation with the axillary CT (*r* = 0.585) in the HT group, followed by the axillary temperature (*r* = 0.558) (Table 3). The forehead temperature had the lowest correlation with the axillary CT (*r* = 0.477). The temperature of the non‐contact trunk surface had a relatively low bias (mean difference, 0.98°C; LoA, 0.19–1.77°C), suggesting that single‐site temperature of the non‐contact trunk surface provided stable and reliable estimates of the axillary CT in the HT group.

**TABLE 3:** Correlation and agreement between axillary CT and NCIT.

NCIT vs. CT	Pearson's correlation coefficient (95% CV)	Agreement	
		Bland–Altman bias (SD)	LoA
HT			
Axilla	0.558 (0.453–0.648)	0.993 (0.402)	(0.206, 1.781)
Forehead	0.477 (0.361–0.579)	1.039 (0.459)	(0.139, 1.938)
Non‐contact trunk	0.585 (0.484–0.670)	0.981 (0.405)	(0.188, 1.774)
Contact trunk	0.493 (0.379–0.593)	1.202 (0.472)	(0.277, 2.126)
Avg. axilla and contact and non‐contact trunk	0.612 (0.516–0.693)	1.058 (0.363)	(0.347, 1.769)
LT			
Axilla	0.410 (0.328–0.492)	0.555 (0.512)	(−0.447, 1.558)
Forehead	0.354 (0.288–0.519)	0.354 (0.487)	(−0.601, 1.309)
Non‐contact trunk	0.483 (0.370–0.583)	0.483 (0.461)	(−0.420, 1.387)
Contact trunk	0.510 (0.399–0.605)	0.862 (0.483)	(−0.085, 1.808)
Avg. axilla and contact and non‐contact trunk	0.586 (0.487–0.670)	0.629 (0.372)	(−0.101, 1.359)
HT + LT			
Axilla	0.512 (0.432–0.588)	0.770 (0.510)	(−0.229, 1.770)
Forehead	0.478 (0.405–0.551)	0.690 (0.584)	(−0.454, 1.835)
Non‐contact trunk	0.484 (0.405–0.563)	0.728 (0.500)	(−0.253, 1.708)
Contact trunk	0.510 (0.432–0.588)	1.029 (0.506)	(0.036, 2.021)
Avg. axilla and contact and non‐contact trunk	0.612 (0.516–0.693)	0.84 (0.425)	(0.006, 1.674)

Abbreviations: Avg., average; CT, contact thermometer; LoA, Limits of agreement; NCIT, Non‐contact infrared thermometer.

The forehead temperature had the lowest correlation with the axillary CT (*r* = 0.354) in the LT and HT groups. The Bland–Altman analysis revealed that no single NCIT measurement consistently agreed with the axillary CT in the LT group. The forehead temperature had the smallest bias (mean difference, 0.35°C; LoA, −0.60 to 1.31°C) in the LT group, but the overall variability was higher than in the HT group (Table 3).

Further analysis indicated that between the HT and LT groups, the older CGA group had a stronger correlation and narrower LoA, particularly at the contact trunk surface in the LT group (Table S1), when stratified by corrected gestational age (CGA).

### Prediction and accuracy of the axillary CT by NCIT‐measured temperature at single or multiple sites

**FIGURE 3:**
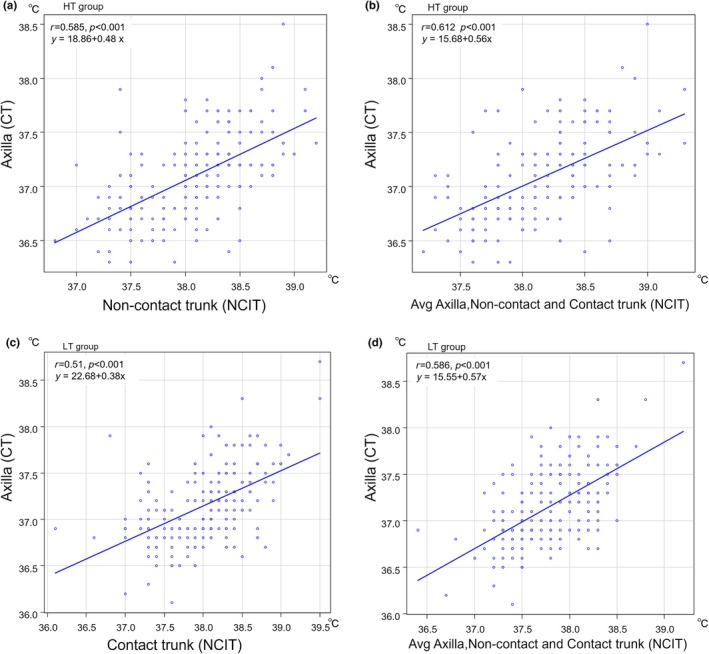
Correlation between the axillary CT and NCIT measurements. Scatter plots illustrate the correlation between the axillary temperature by CT and non‐contact and contact trunk surface temperatures by NCIT (a, c) or the mean of multiple NCIT sites [axilla, non‐contact, and contact trunk surface] (b, d) in HT (a, b) and LT (c, d). Regression lines and corresponding equations, Pearson's correlation coefficients (*r*), and 95% confidence intervals (CIs) are presented.

**FIGURE 4:**
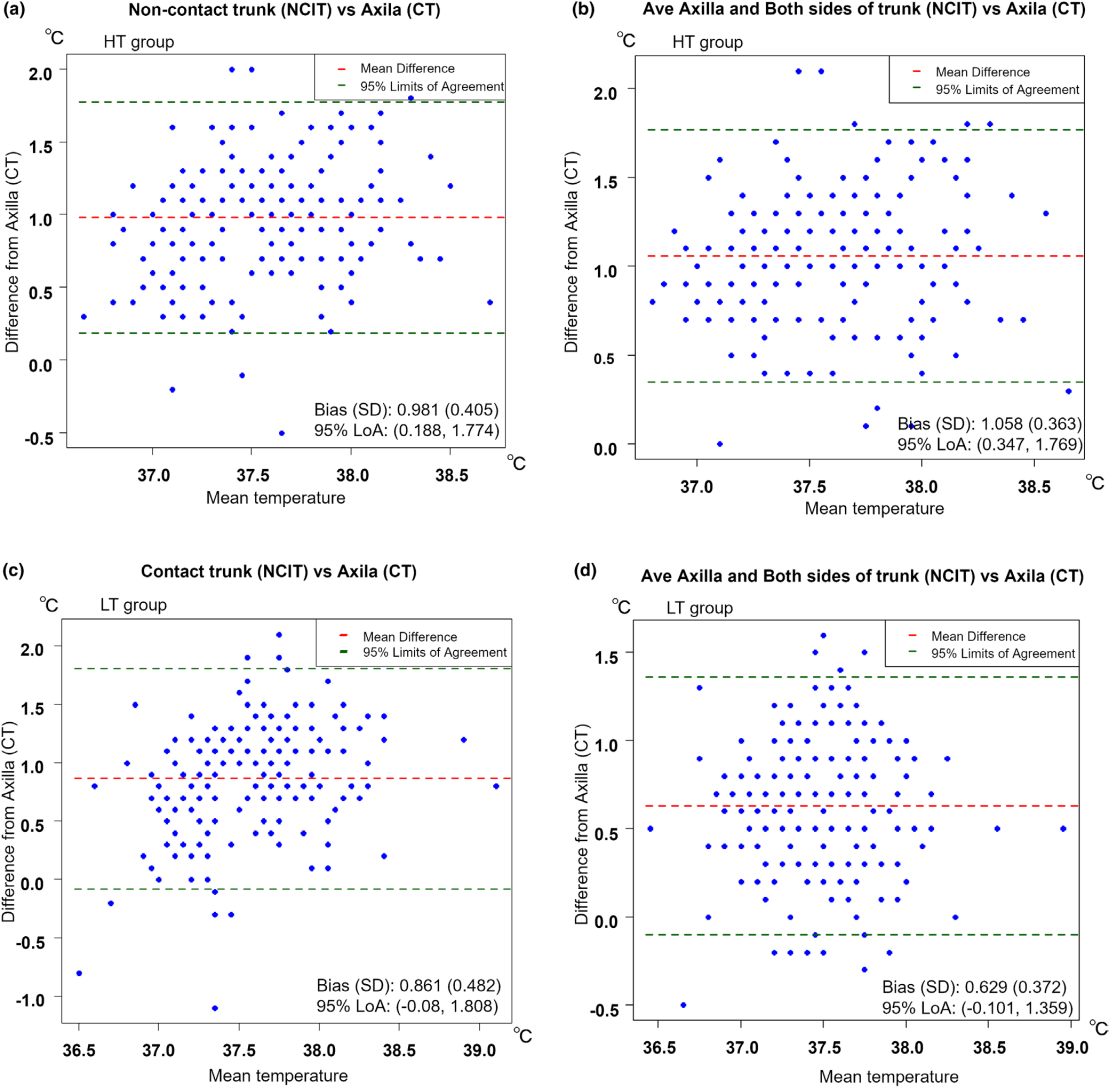
Bland–Altman analysis of AC and NCIT measurements. Bland–Altman plots illustrate the agreement between the axillary CT and non‐contact and contact trunk surface temperatures by NCIT (a, c) or the mean of multiple NCIT sites [axilla and non‐contact and contact trunk surfaces] (b, d) in HT (a, b) and LT (c, d). The mean difference (bias) and standard deviation (SD) are indicated in each plot, along with the 95% limits of agreement (LoA).

Linear regression analysis was performed to determine the prediction equation for the axillary CT using body temperature measured by NCIT. Regression analysis provided the prediction equation for the axillary CT: Y1 using the temperature of non‐contact trunk surface: X1 in the HT group, resulting from the linear regression analysis as follows: Y1 = 18.86 + 0.48 × X1 (*R* ^2^ = 0.342, *p* < 0.001; Figure 3a). The mean values of the axillary, non‐contact, and contact trunk surface temperatures resulted in the highest correlation coefficient in the LT group (*r* = 0.586; Figure 3d) and agreement (mean difference, 0.63°C; LoA, −0.10 to 1.36°C) with the axillary CT rather than each NICT measurement alone (Figure 4d), suggesting that the mean value of multi‐site NCIT may improve measurement reliability under lower incubator temperature conditions. As a result, the prediction equation for the axillary CT: Y2 in the LT group temperatures: X2 resulting from the linear regression analysis was as follows: Y2 = 15.55 + 0.57 × X2 (*R* ^2^ = 0.343, *p* < 0.001; Figure 3d).

### Effects of infant maturity on temperature bias

**FIGURE 5:**
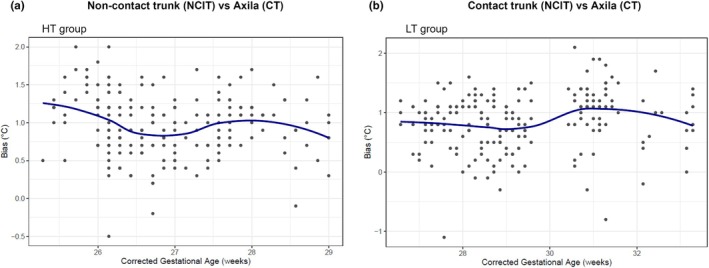
One‐way ANOVA of temperature bias in NCIT measurements. (a) Non‐contact trunk surface (HT group); (b) Contact trunk surface (LT group).

One‐way ANOVA showed that the mean bias was greatest in the subgroup with a CGA ≤26 weeks in the HT group, indicating a limited agreement between NCIT and axillary CT in this population (*p* < 0.01). Similar findings were observed in the LT group at the axillary, forehead, and non‐contact trunk sites, where the bias tended to be larger in infants with a lower CGA (*p* < 0.05). The contact trunk surface showed a relatively stable bias across CGA subgroups <30 weeks, indicating consistent agreement with axillary CT (Figure 5, Tables S2 and S3). Two‐way ANOVA showed no significant interaction between gestational and postnatal age at any site (data not shown).

### Inter‐examiner variability of body temperature at each NICT measurement site

Examiner‐related differences were observed across all measurement sites in the HT group, with ICCs of 4.5%, 7.9%, 6.1%, and 15.6% for axillary, forehead, non‐contact trunk surface, and contact trunk surface, respectively. In contrast, the LT group exhibited less examiner‐related variability, with ICCs of 2.8%, 7.6%, 5.9%, and 0% at the same corresponding sites. Examiner‐dependent variability was consistently greater at the forehead and contact surface sites in the HT group (Table S4).

## DISCUSSION

The current study evaluated NCIT reliability by analyzing the correlation and agreement with AC thermometry under different incubator conditions. NCIT measurements were more stable and correlated with the axillary CT in the HT group, while greater variability and reduced accuracy were observed in the LT group. The Bland–Altman analysis revealed the LoA of NCIT with the axillary CT, especially in the LT group, indicating that the correlation alone does not adequately reflect measurement accuracy. Multi‐site averaging improved the correlation and agreement, supporting the clinical utility for NCIT‐based temperature assessment.

Sollai et al. reported that the mid‐forehead temperature measured by an NCIT demonstrates good agreement with digital axillary thermometers in healthy neonates.^9^ However, few studies have specifically examined the impact of incubator temperature and humidity on NCIT accuracy. The current study demonstrated that NCIT readings are more stable in the HT group, suggesting that a warm and humid environment may contribute to more consistent measurements.

Although NCITs demonstrated a moderate correlation with the axillary CT, the Bland–Altman analysis revealed a wide LoA, especially in the LT group, indicating substantial measurement discrepancies compared to the AC. This finding suggests that the correlation coefficient alone is not sufficient for evaluating NCIT accuracy. Multi‐site averaging significantly improved the correlation and agreement, as evidenced by a narrower LoA when averaging the axillary, non‐contact, and contact surface temperatures. Hamada et al. reported that incubator temperature and humidity influence the measurement accuracy of non‐contact infrared thermography.^7^ In this context, incorporating multiple NCIT measurement sites may enhance reliability, especially when environmental factors introduce greater variability. However, while this approach appears effective in controlled research settings, the clinical practicality and feasibility warrant further investigation.

Morimoto et al. analyzed short‐period fluctuations in neonatal skin temperature (heat oscillations) and reported that larger body weight is associated with greater temperature stability.^16^ Although in the current study neonates in the LT group were more mature and had greater birth weights, the neonates exhibited greater variability in NCIT measurements than neonates in the HT group. Neonates in the LT group may have thicker skin and more developed peripheral blood flow regulation. Under lower temperature and humidity conditions, peripheral vasoconstriction paradoxically tends to occur, leading to greater localized skin temperature fluctuations.^2^, ^7^, ^17^ The difference between body temperature and incubator temperature is thought to have more influence on the variability of NCIT measurements than the weight or maturity of the newborns. In contrast, previous studies have reported that a warm environment enhances skin blood flow and promotes more stable thermoregulation.^3^, ^16^, ^17^ A higher incubator temperature and humidity, thinner skin, and improved peripheral blood circulation likely contributed to more stable temperature readings.

Because a greater temperature bias was observed in younger CGA neonates at most NCIT sites, except for the contact trunk surface in the LT group, the impact of CGA on measurement bias should be considered when using NCIT. Additionally, examiner‐related factors may have influenced NCIT measurement variability in both groups. Because multiple examiners participated in the current study, differences in measurement distance (2–3 cm), angle, and positioning may have contributed to inconsistencies. Moreover, variations in examiner experience could have affected measurement consistency. Owing to large inter‐examiner errors in temperature at the forehead and contact trunk sites under HT environments, standardization of measurement techniques is necessary for reproducibility. While the AC measurements are typically recommended for 5 min, a 30‐s predictive mode is commonly used in NICUs, which may introduce minor discrepancies compared to direct readings. Future studies should focus on standardizing measurement protocols to minimize examiner‐dependent variability.

The lowest correlation efficiency between the forehead temperature and the axillary CT suggests that the forehead temperature is inherently unreliable in the assessment of neonatal temperatures. Uslu et al. also reported that forehead infrared temperature was less reliable.^8^ This finding would be explained by the effects of forehead blood flow regulation, sweating, and the influence of respiratory support devices. Additionally, the small surface area of the forehead may have contributed to measurement variability. Increased sweating due to HT environments and reduced forehead blood flow may have contributed to fluctuations in the forehead temperature, resulting in a weaker correlation with the axillary CT. Robertson‐Smith et al. also observed that neonates receiving bubble CPAP had significantly higher mid‐forehead temperatures compared to axillary temperatures. Respiratory support devices may influence the forehead temperature due to localized heat accumulation from the CPAP interface and humidified air.^13^ Thus, the forehead temperature should be interpreted carefully, especially in neonates receiving assisted ventilation.

Duran et al. reported that contact axillary temperature measurement in NICUs leads to significantly higher Premature Infant Pain Profile (PIPP) scores compared to other methods, indicating increased procedural stress.^18^ Carbajal et al. emphasized that the high frequency of painful procedures in NICUs has a potential neurodevelopmental impact.^4^ NCITs minimize unnecessary physical contact, contributing to a less stressful environment for neonates and reducing opportunities for contact infection, which may affect accuracy depending on the incubator setting. While infrared thermography is expensive and requires specialized analysis, NCITs are cost‐effective and suitable for routine NICU use.

The current study had several limitations. First, as a single‐center observational study, the reproducibility in other NICU settings has yet to be verified. Multi‐center validation is needed to confirm these findings. Second, although NCIT accuracy may be influenced by skin status and peripheral circulatory dynamics, the current study did not perform a detailed analysis of these factors. Future studies should incorporate a detailed analysis of the effects of skin blood flow and humidity to assess the accuracy of NCIT for neonates with a variety of diseases. To improve the accuracy of NCITs and make NCITs clinically useful, it would be beneficial to develop regression‐based correction models tailored to specific environmental conditions.

## CONCLUSIONS

NCITs serve as a viable, non‐invasive alternative to conventional CTs in neonatal care. NCITs provide reliable temperature readings with minimal stress to neonates, although proper selection of the measurement site is essential to ensure accuracy.

Future research should focus on improving calibration methods for different measurement sites and environmental conditions to enhance the accuracy and clinical utility of NCITs in various NICU settings.

## AUTHOR CONTRIBUTIONS

TO conceptualized and designed the study, conducted the initial analysis, drafted the initial manuscript, and reviewed and revised the manuscript. YO contributed to the study conceptualization and design, drafted the initial manuscript, and reviewed and revised the manuscript. TS and AI critically reviewed the manuscript for important intellectual content. All authors fulfilled the authorship criteria, approved the final manuscript, and agreed to take responsibility for all aspects of the study.

## CONFLICT OF INTEREST STATEMENT

The authors declare no conflicts of interest.

## Supporting information


Table S1.

## Data Availability

The data that support the findings of this study are available on request from the corresponding author. The data are not publicly available due to privacy or ethical restrictions.
