# Supplementary material for: Utility of non‐contact thermometers in neonatal intensive care: Effects of incubator conditions and measurement sites
Source: Pediatr Int. 2025 Oct 14;67(1):e70231. doi: 10.1111/ped.70231 (PMC12519928; doi:10.1111/ped.70231)
Supplement: Supplementary file 1 — Table S1. [file PED-67-e70231-s001.docx]

Table S1. Correlation between NCIT measurements and axillary CT

Stratified by CGA ≤ 26.8 vs. > 26.8 weeks in the HT group; CGA ≤ 29 vs. > 29 weeks in the LT group

| NCIT vs. CT CGA | | Pearson’s correlation coefficient | Agreement | |
| --- | --- | --- | --- | --- |
|  |  |  | Bland-Altman Bias (SD) | LoA |
| HT median = 26.8 weeks | |  |  |  |
| Axilla | ≤ Median | 0.537 | 0.960 (0.407) | (0.163, 1.757) |
|  | > Median | 0.581 | 1.027 (0.396) | (0.251, 1.803) |
| Forehead | ≤ Median | 0.500 | 1.157 (0.432) | (0.311, 2.003) |
|  | > Median | 0.489 | 0.921 (0.457) | (0.025, 1.816) |
| Non-contact trunk | ≤ Median | 0.528 | 1.012 (0.456) | (0.119, 1.906) |
|  | > Median | 0.660 | 0.949 (0.346) | (0.272, 1.627) |
| Contact trunk | ≤ Median | 0.445 | 1.247 (0.510) | (0.249, 2.246) |
|  | > Median | 0.554 | 1.156 (0.428) | (0.316, 1.995) |
| LT median = 29 weeks | |  |  |  |
| Axilla | ≤ Median | 0.420 | 0.529 (0.491) | (-0.433, 1.492) |
|  | > Median | 0.403 | 0.582 (0.533) | (-0.463, 1.627) |
| Forehead | ≤ Median | 0.467 | 0.397 (0.492) | (-0.567, 1.361) |
|  | > Median | 0.483 | 0.310 (0.481) | (-0.633, 1.253) |
| Non-contact trunk | ≤ Median | 0.446 | 0.535 (0.451) | (-0.348, 1.419) |
|  | > Median | 0.510 | 0.429 (0.467) | (-0.487, 1.345) |
| Contact trunk | ≤ Median | 0.473 | 0.757 (0.448) | (-0.09, 1.665) |
|  | > Median | 0.558 | 0.938 (0.508) | (-0.059, 1.933) |

Abbreviations: NCIT, non-contact infrared thermometer; CGA, corrected gestational age; CT, contact thermometer; LoA, limits of agreement

Table S2. Bias analysis by corrected gestational age (HT group)

| Measurement site (NCIT) | Corrected GA | Mean Bias* (℃) | SD(℃) | n | *P*-value |
| --- | --- | --- | --- | --- | --- |
| Axilla | ≤ 26w 0d | 1.09 | 0.401 | 30 | 0.0476 |
|  | 26w 1d – 27w 0d | 0.912 | 0.424 | 89 |  |
|  | 27w 1d – 27w 6d | 1.10 | 0.321 | 37 |  |
|  | ≥ 28w 0d | 1.00 | 0.392 | 38 |  |
| Forehead | ≤ 26w 0d | 1.39 | 0.379 | 30 | 0.0000264 |
|  | 26w 1d – 27w 0d | 0.964 | 0.471 | 89 |  |
|  | 27w 1d – 27w 6d | 0.908 | 0.395 | 37 |  |
|  | ≥ 28w 0d | 1.07 | 0.421 | 38 |  |
| Non-contact trunk | ≤ 26w 0d | 1.26 | 0.365 | 30 | 0.000278 |
|  | 26w 1d – 27w 0d | 0.896 | 0.423 | 89 |  |
|  | 27w 1d – 27w 6d | 0.943 | 0.341 | 37 |  |
|  | ≥ 28w 0d | 1.00 | 0.362 | 38 |  |
| Contact trunk | ≤ 26w 0d | 1.56 | 0.422 | 30 | 0.0000133 |
|  | 26w 1d – 27w 0d | 1.07 | 0.492 | 89 |  |
|  | 27w 1d – 27w 6d | 1.19 | 0.264 | 37 |  |
|  | ≥ 28w 0d | 1.24 | 0.484 | 38 |  |

Abbreviations: NCIT, non-contact infrared thermometer; GA, gestational age

* Mean bias = (NCIT temperature) − (axillary temperature using contact thermometer)

All *P*-values were obtained using a one-way ANOVA that compared the mean biases across the four CGA strata.

Table S3. Bias analysis by corrected gestational age (LT group)

| Measurement site (NCIT) | Corrected GA | Mean Bias (℃) | SD(℃) | n | *P*-value |
| --- | --- | --- | --- | --- | --- |
| Axilla | ≤ 27w 0d | 0.860 | 0.392 | 10 | 0.0005 |
|  | 27w 1d–28w 0d | 0.702 | 0.354 | 41 |  |
|  | 28w 1d–29w 0d | 0.325 | 0.520 | 51 |  |
|  | 29w 1d–30w 0d | 0.475 | 0.513 | 28 |  |
|  | ≥ 30w 1d | 0.624 | 0.539 | 71 |  |
| Forehead | ≤ 27w 0d | 0.730 | 0.422 | 10 | 0.0002 |
|  | 27w 1d–28w 0d | 0.561 | 0.450 | 41 |  |
|  | 28w 1d–29w 0d | 0.200 | 0.459 | 51 |  |
|  | 29w 1d–30w 0d | 0.207 | 0.445 | 28 |  |
|  | ≥ 30w 1d | 0.351 | 0.491 | 71 |  |
| Non-contact trunk | ≤ 27w 0d | 0.740 | 0.331 | 10 | 0.0330 |
|  | 27w 1d–28w 0d | 0.639 | 0.452 | 41 |  |
|  | 28w 1d–29w 0d | 0.412 | 0.441 | 51 |  |
|  | 29w 1d–30w 0d | 0.429 | 0.395 | 28 |  |
|  | ≥ 30w 1d | 0.430 | 0.496 | 71 |  |
| Contact trunk | ≤ 27w 0d | 0.780 | 0.377 | 10 | 0.0237 |
|  | 27w 1d–28w 0d | 0.827 | 0.455 | 41 |  |
|  | 28w 1d–29w 0d | 0.757 | 0.460 | 51 |  |
|  | 29w 1d–30w 0d | 0.754 | 0.414 | 28 |  |
|  | ≥ 30w 1d | 1.010 | 0.525 | 71 |  |

Abbreviations: NCIT, non-contact infrared thermometer; GA, gestational age

* Mean bias = (NCIT temperature) − (axillary temperature using contact thermometer)

All *P*-values were obtained using a one-way ANOVA that compared the mean biases across the five CGA strata.

Table S4. Inter-examiner variability by NCIT site and group

| Measurement site (NCIT) | Group | Examiner Variance | Residual Variance | ICC* (%) |
| --- | --- | --- | --- | --- |
| Axilla | Overall (n = 395) | 0.00067 | 0.22720 | 0.29 |
|  | HT (n = 194) | 0.00645 | 0.13627 | 4.5 |
|  | LT (n = 201) | 0.00632 | 0.22043 | 2.8 |
| Forehead | Overall | 0.01977 | 0.29020 | 6.4 |
|  | HT | 0.01480 | 0.17340 | 7.9 |
|  | LT | 0.01649 | 0.19991 | 7.6 |
| Non-contact trunk | Overall | 0.00928 | 0.21688 | 4.1 |
|  | HT | 0.00936 | 0.14389 | 6.1 |
|  | LT | 0.01095 | 0.17550 | 5.9 |
| Contact trunk | Overall | 0.00801 | 0.22828 | 3.4 |
|  | HT | 0.03270 | 0.17720 | 15.6 |
|  | LT | 0.00000 | 0.21780 | 0.0 |

Abbreviations: NCIT, non-contact infrared thermometer; ICC, intraclass correlation coefficient

* ICC = examiner variance / (examiner variance + residual variance)
